# Bis(4-carb­oxy­pyridine-2-carboxyl­ato-κ^2^ *N*,*O* ^2^)copper(II) dimethyl sulfoxide disolvate

**DOI:** 10.1107/S1600536811003424

**Published:** 2011-01-29

**Authors:** Hossein Aghabozorg, Saba Goodarzi, Masoud Mirzaei, Behrouz Notash

**Affiliations:** a Faculty of Chemistry, Islamic Azad University, North Tehran Branch, Tehran, Iran; b Department of Chemistry, School of Sciences, Ferdowsi University of Mashhad, Mashhad, Iran; c Department of Chemistry, Shahid Beheshti University, G.C., Evin, Tehran 1983963113, Iran

## Abstract

In the title complex, [Cu(C_7_H_4_NO_4_)_2_]·2C_2_H_6_OS, the Cu^II^ atom is situated on an inversion centre and is *N*,*O*-chelated by two monoanionic 4-carb­oxy­pyridine-2-carboxyl­ate ligands in a slightly distorted square-planar coordination geometry. The dimethyl sulfoxide solvent mol­ecules and Cu^II^ complex mol­ecules are linked by O—H⋯O hydrogen bonding. In addition, C—H⋯O contacts and π–π inter­actions [centroid–centroid distance = 3.590 (1) Å] occur.

## Related literature

For the design and synthesis of coordination compounds and complexes derived from pyridine-2,4-dicarb­oxy­lic acid, see: Aghabozorg *et al.* (2008 ▶); Noro *et al.* (2005 ▶).
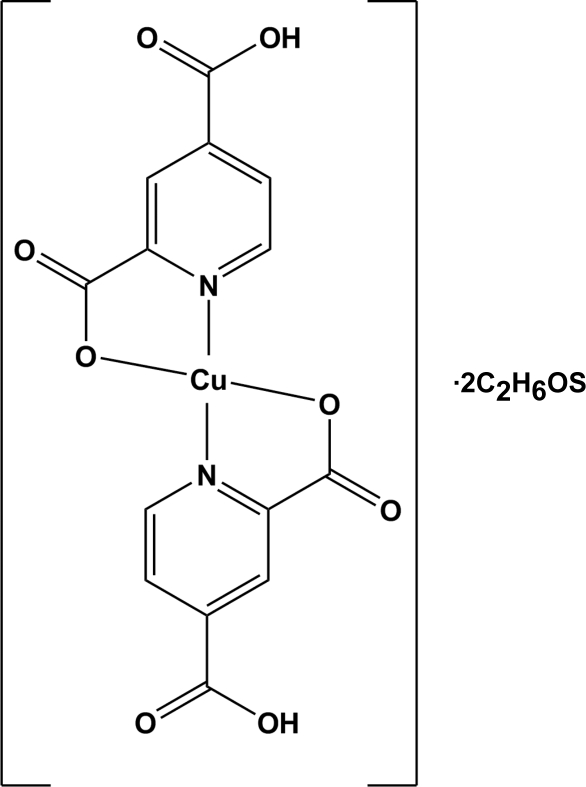


## Experimental

### 

#### Crystal data

[Cu(C_7_H_4_NO_4_)_2_]·2C_2_H_6_OS
*M* *_r_* = 552.05
Triclinic, 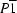

*a* = 6.8831 (14) Å
*b* = 7.5218 (15) Å
*c* = 11.719 (2) Å
α = 102.95 (3)°
β = 91.86 (3)°
γ = 111.12 (3)°
*V* = 547.3 (2) Å^3^
*Z* = 1
Mo *K*α radiation
μ = 1.25 mm^−1^
*T* = 298 K
0.2 × 0.10 × 0.05 mm

#### Data collection

Stoe IPDS II diffractometer
6125 measured reflections
2928 independent reflections
2428 reflections with *I* > 2σ(*I*)
*R*_int_ = 0.034

#### Refinement

*R*[*F* ^2^ > 2σ(*F* ^2^)] = 0.040
*wR*(*F* ^2^) = 0.092
*S* = 1.08
2928 reflections
157 parameters
H atoms treated by a mixture of independent and constrained refinement
Δρ_max_ = 0.38 e Å^−3^
Δρ_min_ = −0.30 e Å^−3^

### 

Data collection: *X-AREA* (Stoe & Cie, 2005 ▶); cell refinement: *X-AREA*; data reduction: *X-AREA*; program(s) used to solve structure: *SHELXS97* (Sheldrick, 2008 ▶); program(s) used to refine structure: *SHELXL97* (Sheldrick, 2008 ▶); molecular graphics: *ORTEP-3 for Windows* (Farrugia, 1997 ▶); software used to prepare material for publication: *WinGX* (Farrugia, 1999 ▶).

## Supplementary Material

Crystal structure: contains datablocks I, global. DOI: 10.1107/S1600536811003424/bt5466sup1.cif

Structure factors: contains datablocks I. DOI: 10.1107/S1600536811003424/bt5466Isup2.hkl

Additional supplementary materials:  crystallographic information; 3D view; checkCIF report

## Figures and Tables

**Table 1:** Hydrogen-bond geometry (Å, °)

*D*—H⋯*A*	*D*—H	H⋯*A*	*D*⋯*A*	*D*—H⋯*A*
O3—H3⋯O5^i^	0.84 (4)	1.68 (4)	2.518 (3)	173 (4)
C4—H4⋯O3^ii^	0.93	2.55	3.427 (3)	158
C5—H5⋯O5^iii^	0.93	2.55	3.370 (3)	147
C8—H8*B*⋯O2^iv^	0.96	2.38	3.223 (3)	147
C9—H9*C*⋯O4	0.96	2.51	3.448 (4)	164

Symmetry codes: (i) 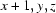; (ii) 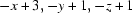; (iii) 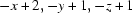; (iv) 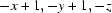.

**Fig. 1.**
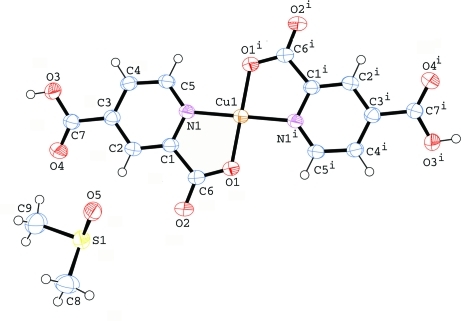
The molecular structure of the title compound with displacement ellipsoids drawn at 50% probability level (symmetry code: i: 1 - x,-y,1 - z).

**Fig. 2.**
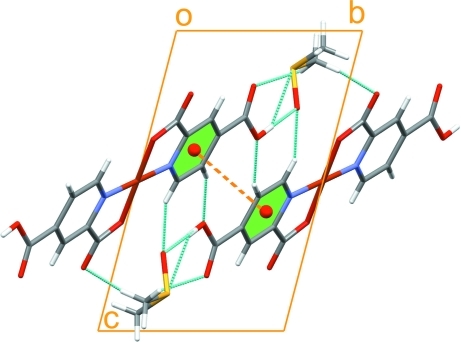
Packing diagram of the title compound. The intermolecular O—H···O, and C—H···O hydrogen bonds and π···π contacts are shown as blue and orange dashed lines, respectively.

## supplementary crystallographic information

### Comment

Great interest has been focused on the rapidly expanding field of supramolecular chemistry and crystal engineering of the coordination compounds in recent years because of their intriguing network topologies as well as their potential application as functional materials in many areas (Aghabozorg *et al.*, 2008). Pyridine-2,4-dicarboxylic acid (2,4-pydcH_2_) is a good building block for constructing   complexes. However, plenty of researches have focused on the supramolecular chemistry and coordination polymers which only include single carboxylic acid ligands, (Noro *et al.*, 2005). In this paper, we report the crystal structure of the title compound prepared from Cu(NO_3_)_2_.3H_2_O, 2,4-pydcH_2_ and acridine.

The structure of title complex is shown in Fig. 1. In the complex, 2,4-pydcH ligands are bound to one Cu^II^ ion through pyridine N and deprotonated carboxylate O atoms at 2-positions, leading to a distorted square planar geometry around the metal ion. The carboxylic groups at the 4-position of 2,4-pydcH ligands are not coordinating. [Cu(C_14_H_8_N_2_O_8_)] complex is connected into two-dimensional layers through H-bonding interactions (Table 1).  The crystal packing is additionally stabilized by π-π stacking interactions (Fig. 2).

### Experimental

A mixture of 2,4-pydcH_2_ (83 mg, 0.50 mmol), Cu(NO_3_)_2_.3H_2_O (120 mg, 0.50 mmol), acridine (179 mg, 1.0 mmol) in 18 ml me thanol/DMSO were heated and stirred for 2 hrs, and then cooled to room temperature. The reaction yielded purple plate crystals of the title compound after 2 months.

### Refinement

The hydrogen atoms of the carboxylic acid group was found in a difference Fourier map and refined isotropically without restraint. The C—H protons were positioned geometrically and refined as riding atoms with C—H = 0.93 Å and *U*iso(H) = 1.2 *U*eq(C) for aromatic C—H groups and C—H = 0.96 Å and *U*iso(H) = 1.5 *Ueq*(C) for the methyl groups.

### Crystal data

**Table:**

[Cu(C_7_H_4_NO_4_)_2_]·2C_2_H_6_OS	*Z* = 1
*M**_r_* = 552.05	*F*(000) = 283
Triclinic, *P*1	*D*_x_ = 1.675 Mg m^−^^3^
Hall symbol: -P 1	Mo *K*α radiation, λ = 0.71073 Å
*a* = 6.8831 (14) Å	Cell parameters from 2928 reflections
*b* = 7.5218 (15) Å	θ = 3.0–29.1°
*c* = 11.719 (2) Å	µ = 1.25 mm^−^^1^
α = 102.95 (3)°	*T* = 298 K
β = 91.86 (3)°	Plate, purple
γ = 111.12 (3)°	0.2 × 0.1 × 0.05 mm
*V* = 547.3 (2) Å^3^	

### Data collection

**Table:**

Stoe IPDS II diffractometer	2428 reflections with *I* > 2σ(*I*)
Radiation source: fine-focus sealed tube	*R*_int_ = 0.034
graphite	θ_max_ = 29.1°, θ_min_ = 3.0°
Detector resolution: 0.15 mm pixels mm^-1^	*h* = −9→9
rotation method scans	*k* = −10→10
6125 measured reflections	*l* = −16→15
2928 independent reflections	

### Refinement

**Table:**

Refinement on *F*^2^	Primary atom site location: structure-invariant direct methods
Least-squares matrix: full	Secondary atom site location: difference Fourier map
*R*[*F*^2^ > 2σ(*F*^2^)] = 0.040	Hydrogen site location: inferred from neighbouring sites
*wR*(*F*^2^) = 0.092	H atoms treated by a mixture of independent and constrained refinement
*S* = 1.08	*w* = 1/[σ^2^(*F*_o_^2^) + (0.0365*P*)^2^ + 0.2725*P*] where *P* = (*F*_o_^2^ + 2*F*_c_^2^)/3
2928 reflections	(Δ/σ)_max_ = 0.002
157 parameters	Δρ_max_ = 0.38 e Å^−^^3^
0 restraints	Δρ_min_ = −0.30 e Å^−^^3^

### Special details

**Table:**

Geometry. All e.s.d.'s (except the e.s.d. in the dihedral angle between two l.s. planes) are estimated using the full covariance matrix. The cell e.s.d.'s are taken into account individually in the estimation of e.s.d.'s in distances, angles and torsion angles; correlations between e.s.d.'s in cell parameters are only used when they are defined by crystal symmetry. An approximate (isotropic) treatment of cell e.s.d.'s is used for estimating e.s.d.'s involving l.s. planes.
Refinement. Refinement of *F*^2^ against ALL reflections. The weighted *R*-factor *wR* and goodness of fit *S* are based on *F*^2^, conventional *R*-factors *R* are based on *F*, with *F* set to zero for negative *F*^2^. The threshold expression of *F*^2^ > σ(*F*^2^) is used only for calculating *R*-factors(gt) *etc*. and is not relevant to the choice of reflections for refinement. *R*-factors based on *F*^2^ are statistically about twice as large as those based on *F*, and *R*- factors based on ALL data will be even larger.

### Fractional atomic coordinates and isotropic or equivalent isotropic displacement parameters (Å^2^)

**Table:**

	*x*	*y*	*z*	*U*_iso_*/*U*_eq_	
O5	0.7477 (3)	0.7579 (3)	0.25725 (15)	0.0572 (5)	
S1	0.74093 (10)	0.67910 (10)	0.12483 (5)	0.04660 (16)	
Cu1	0.5000	0.0000	0.5000	0.03607 (12)	
O1	0.3689 (2)	0.0279 (3)	0.36230 (14)	0.0448 (4)	
C6	0.4947 (3)	0.1284 (4)	0.30111 (19)	0.0383 (5)	
C2	0.8846 (3)	0.3107 (3)	0.29424 (18)	0.0362 (4)	
H2	0.8551	0.3418	0.2249	0.043*	
C1	0.7250 (3)	0.2025 (3)	0.34780 (18)	0.0339 (4)	
O2	0.4440 (3)	0.1668 (3)	0.21140 (16)	0.0540 (5)	
N1	0.7628 (3)	0.1553 (3)	0.44860 (15)	0.0331 (4)	
C7	1.2654 (4)	0.4892 (4)	0.28672 (19)	0.0387 (5)	
C5	0.9603 (3)	0.2151 (3)	0.49832 (18)	0.0358 (4)	
H5	0.9857	0.1825	0.5678	0.043*	
C4	1.1283 (3)	0.3242 (3)	0.44914 (19)	0.0368 (4)	
H4	1.2648	0.3647	0.4853	0.044*	
C3	1.0910 (3)	0.3727 (3)	0.34541 (19)	0.0355 (4)	
O3	1.4430 (3)	0.5841 (3)	0.35694 (15)	0.0481 (4)	
O4	1.2395 (3)	0.4936 (3)	0.18477 (15)	0.0526 (5)	
C8	0.6304 (5)	0.8138 (5)	0.0578 (2)	0.0612 (7)	
H8A	0.4882	0.7847	0.0744	0.092*	
H8B	0.6323	0.7782	−0.0260	0.092*	
H8C	0.7104	0.9522	0.0883	0.092*	
C9	1.0046 (5)	0.7850 (6)	0.0982 (3)	0.0713 (9)	
H9A	1.0577	0.9246	0.1323	0.107*	
H9B	1.0111	0.7597	0.0147	0.107*	
H9C	1.0879	0.7280	0.1332	0.107*	
H3	1.538 (6)	0.645 (5)	0.320 (3)	0.076 (11)*	

### Atomic displacement parameters (Å^2^)

**Table:**

	*U*^11^	*U*^22^	*U*^33^	*U*^12^	*U*^13^	*U*^23^
O5	0.0428 (9)	0.0775 (13)	0.0357 (9)	0.0011 (9)	0.0015 (7)	0.0208 (9)
S1	0.0437 (3)	0.0481 (4)	0.0400 (3)	0.0059 (3)	0.0025 (2)	0.0150 (3)
Cu1	0.03021 (19)	0.0481 (2)	0.03117 (19)	0.01310 (16)	0.00208 (14)	0.01565 (16)
O1	0.0315 (8)	0.0623 (11)	0.0408 (8)	0.0127 (7)	−0.0007 (6)	0.0226 (8)
C6	0.0351 (11)	0.0454 (12)	0.0332 (10)	0.0140 (9)	−0.0016 (8)	0.0110 (9)
C2	0.0378 (11)	0.0411 (11)	0.0286 (9)	0.0131 (9)	−0.0010 (8)	0.0105 (8)
C1	0.0341 (10)	0.0378 (11)	0.0288 (9)	0.0133 (8)	−0.0010 (8)	0.0077 (8)
O2	0.0437 (9)	0.0719 (12)	0.0461 (10)	0.0140 (9)	−0.0067 (7)	0.0293 (9)
N1	0.0336 (8)	0.0394 (9)	0.0272 (8)	0.0142 (7)	0.0019 (6)	0.0096 (7)
C7	0.0362 (11)	0.0479 (13)	0.0343 (10)	0.0167 (10)	0.0045 (8)	0.0134 (9)
C5	0.0359 (10)	0.0437 (12)	0.0296 (9)	0.0161 (9)	−0.0002 (8)	0.0117 (8)
C4	0.0321 (10)	0.0452 (12)	0.0324 (10)	0.0149 (9)	−0.0008 (8)	0.0089 (9)
C3	0.0354 (10)	0.0402 (11)	0.0302 (9)	0.0146 (9)	0.0032 (8)	0.0072 (8)
O3	0.0342 (8)	0.0658 (12)	0.0363 (8)	0.0073 (8)	0.0045 (7)	0.0169 (8)
O4	0.0450 (10)	0.0746 (13)	0.0379 (9)	0.0159 (9)	0.0047 (7)	0.0249 (9)
C8	0.0671 (18)	0.079 (2)	0.0426 (14)	0.0313 (16)	0.0007 (13)	0.0185 (14)
C9	0.0473 (16)	0.109 (3)	0.0604 (18)	0.0251 (17)	0.0136 (14)	0.0325 (18)

### Geometric parameters (Å, °)

**Table:**

O5—S1	1.5247 (19)	C7—O4	1.212 (3)
S1—C8	1.760 (3)	C7—O3	1.313 (3)
S1—C9	1.770 (3)	C7—C3	1.499 (3)
Cu1—O1	1.9123 (16)	C5—C4	1.384 (3)
Cu1—O1^i^	1.9123 (16)	C5—H5	0.9300
Cu1—N1	1.9657 (19)	C4—C3	1.387 (3)
Cu1—N1^i^	1.9657 (19)	C4—H4	0.9300
O1—C6	1.284 (3)	O3—H3	0.84 (4)
C6—O2	1.223 (3)	C8—H8A	0.9600
C6—C1	1.514 (3)	C8—H8B	0.9600
C2—C1	1.376 (3)	C8—H8C	0.9600
C2—C3	1.392 (3)	C9—H9A	0.9600
C2—H2	0.9300	C9—H9B	0.9600
C1—N1	1.351 (3)	C9—H9C	0.9600
N1—C5	1.334 (3)		
			
O5—S1—C8	105.52 (14)	O3—C7—C3	113.21 (19)
O5—S1—C9	104.03 (14)	N1—C5—C4	121.70 (19)
C8—S1—C9	99.68 (17)	N1—C5—H5	119.2
O1—Cu1—O1^i^	180.00 (5)	C4—C5—H5	119.2
O1—Cu1—N1	84.57 (7)	C5—C4—C3	119.3 (2)
O1^i^—Cu1—N1	95.43 (7)	C5—C4—H4	120.4
O1—Cu1—N1^i^	95.43 (7)	C3—C4—H4	120.4
O1^i^—Cu1—N1^i^	84.57 (7)	C4—C3—C2	118.7 (2)
N1—Cu1—N1^i^	180.0	C4—C3—C7	122.2 (2)
C6—O1—Cu1	115.22 (14)	C2—C3—C7	119.08 (19)
O2—C6—O1	125.9 (2)	C7—O3—H3	111 (2)
O2—C6—C1	119.3 (2)	S1—C8—H8A	109.5
O1—C6—C1	114.78 (18)	S1—C8—H8B	109.5
C1—C2—C3	118.97 (19)	H8A—C8—H8B	109.5
C1—C2—H2	120.5	S1—C8—H8C	109.5
C3—C2—H2	120.5	H8A—C8—H8C	109.5
N1—C1—C2	121.90 (19)	H8B—C8—H8C	109.5
N1—C1—C6	114.33 (19)	S1—C9—H9A	109.5
C2—C1—C6	123.77 (18)	S1—C9—H9B	109.5
C5—N1—C1	119.41 (19)	H9A—C9—H9B	109.5
C5—N1—Cu1	129.51 (15)	S1—C9—H9C	109.5
C1—N1—Cu1	111.07 (14)	H9A—C9—H9C	109.5
O4—C7—O3	124.8 (2)	H9B—C9—H9C	109.5
O4—C7—C3	122.0 (2)		
			
N1—Cu1—O1—C6	−0.79 (18)	O1^i^—Cu1—N1—C5	0.0 (2)
N1^i^—Cu1—O1—C6	179.21 (18)	O1—Cu1—N1—C1	−0.07 (15)
Cu1—O1—C6—O2	−178.7 (2)	O1^i^—Cu1—N1—C1	179.93 (15)
Cu1—O1—C6—C1	1.4 (3)	C1—N1—C5—C4	−0.1 (3)
C3—C2—C1—N1	0.1 (3)	Cu1—N1—C5—C4	179.81 (16)
C3—C2—C1—C6	179.5 (2)	N1—C5—C4—C3	−0.1 (3)
O2—C6—C1—N1	178.6 (2)	C5—C4—C3—C2	0.4 (3)
O1—C6—C1—N1	−1.5 (3)	C5—C4—C3—C7	−179.5 (2)
O2—C6—C1—C2	−0.8 (4)	C1—C2—C3—C4	−0.4 (3)
O1—C6—C1—C2	179.2 (2)	C1—C2—C3—C7	179.5 (2)
C2—C1—N1—C5	0.1 (3)	O4—C7—C3—C4	162.8 (2)
C6—C1—N1—C5	−179.27 (19)	O3—C7—C3—C4	−17.7 (3)
C2—C1—N1—Cu1	−179.83 (17)	O4—C7—C3—C2	−17.1 (4)
C6—C1—N1—Cu1	0.8 (2)	O3—C7—C3—C2	162.4 (2)
O1—Cu1—N1—C5	180.0 (2)		

Symmetry codes: (i) −*x*+1, −*y*, −*z*+1.

### Hydrogen-bond geometry (Å, °)

**Table:**

*D*—H···*A*	*D*—H	H···*A*	*D*···*A*	*D*—H···*A*
O3—H3···O5^ii^	0.84 (4)	1.68 (4)	2.518 (3)	173 (4)
C4—H4···O3^iii^	0.93	2.55	3.427 (3)	158
C5—H5···O5^iv^	0.93	2.55	3.370 (3)	147
C8—H8B···O2^v^	0.96	2.38	3.223 (3)	147
C9—H9C···O4	0.96	2.51	3.448 (4)	164

Symmetry codes: (ii) *x*+1, *y*, *z*; (iii) −*x*+3, −*y*+1, −*z*+1; (iv) −*x*+2, −*y*+1, −*z*+1; (v) −*x*+1, −*y*+1, −*z*.
